# The consistency of categorization-consistency in speech perception

**DOI:** 10.3758/s13423-025-02700-x

**Published:** 2025-04-24

**Authors:** Hyoju Kim, Bob McMurray, Eldon Sorensen, Jacob Oleson

**Affiliations:** 1 Department of Psychological and Brain Sciences, University of Iowa, 255E PBSB, Iowa City, IA 52242 USA; 2 Department of Linguistics, University of Iowa, Iowa City, IA USA; 3 Sandia National Laboratories, Albuquerque, NM USA; 4 Department of Biostatistics, University of Iowa, Iowa City, IA USA

**Keywords:** Speech perception, Categorization consistency, Individual differences, Visual analog scaling

## Abstract

Listeners generally map continuous acoustic information onto categories in a gradient manner with varying individual differences. Typically, such individual differences in speech categorization have been characterized by the mean slope of the response function, as quantified through the visual analog scaling (VAS) task. However, recent evidence suggests that categorization consistency (trial-by-trial response variability) may be a more robust predictor of real-world outcomes than the mean slope. Despite this, the extent to which these VAS indices represent reliable and stable traits relevant to speech perception remains uncertain. This study investigates the stability and trait-like nature of VAS indices by analyzing VAS responses across a diverse range of speech continua. We also examine the potential associations between differences in speech categorization and broader cognitive differences. American English-speaking adults (*n* = 68) completed the VAS task, alongside three questionnaires assessing autistic traits, anxiety, and impulsivity. We found that categorization consistency showed stronger correlations across continuum types than the categorization slope. Furthermore, no significant correlations were observed between the VAS indices and broader cognitive factors. These results suggest categorization consistency is a more stable property of individuals over the mean slope, and importantly, it is not an artifact due to higher cognitive factors. Thus, the extent to which listeners are consistent in their speech categorization may provide a more accurate characterization of individual differences in speech perception.

**Supplementary information:**

The online version contains supplementary material available at 10.3758/s13423-025-02700-x.

## Introduction

Listeners must categorize incoming speech into categories, like /d/ and /t/, to accurately recognize words and their meanings. However, speech categorization is complex due to inherent variability in how these categories are instantiated, which arises from differences among talkers, speaking rates, and contexts (McMurray & Jongman, 2011). Given its importance to downstream language processing, many studies have used measures of speech categorization to examine individual differences and differences in clinical populations. These studies have linked differences in speech categorization to clinical concerns like developmental language disorder (DLD; Coady et al., 2005; Robertson et al., 2009) and dyslexia (Serniclaes et al., 2004; Werker & Tees, 1987), to differences in populations like multilinguals (Casillas, 2015, 2020; Flege et al., 1999; Pan et al., 2022) or to differences as a function of development (Hazan & Barrett, 2000; Nittrouer, 2002) and aging (DiNino, 2024; Toscano & Lansing, 2019).

Although these studies have often found differences in low-level speech categorization, their findings are not always consistent, even within a population such as people with DLD (e.g., Coady et al., 2005, 2007; McMurray et al., 2014; Robertson et al., 2009). This raises the possibility of methodological concerns. Importantly, as we describe, traditional forced-choice measures leave a fundamental ambiguity about the nature of the underlying traits and what precisely differs among people (McMurray, 2022). In these forced-choice tasks, most or all of the above-mentioned groups look similar—they show a shallower slope than “modal” listeners. However, this coarse picture may mask important mechanistic differences in how speech perception differs across these groups and the potential different developmental origins of these differences. This raises the need for more sensitive tasks.

An emerging task—the visual analog scaling (VAS) task (Apfelbaum et al., 2022)—may offer a more promising psychometric footing for research on individual differences. Thus, as part of a larger effort to examine these population differences, the present study starts to unpack some of the critical psychometric properties of this task.

### Measuring speech categorization

**Fig. 1:**
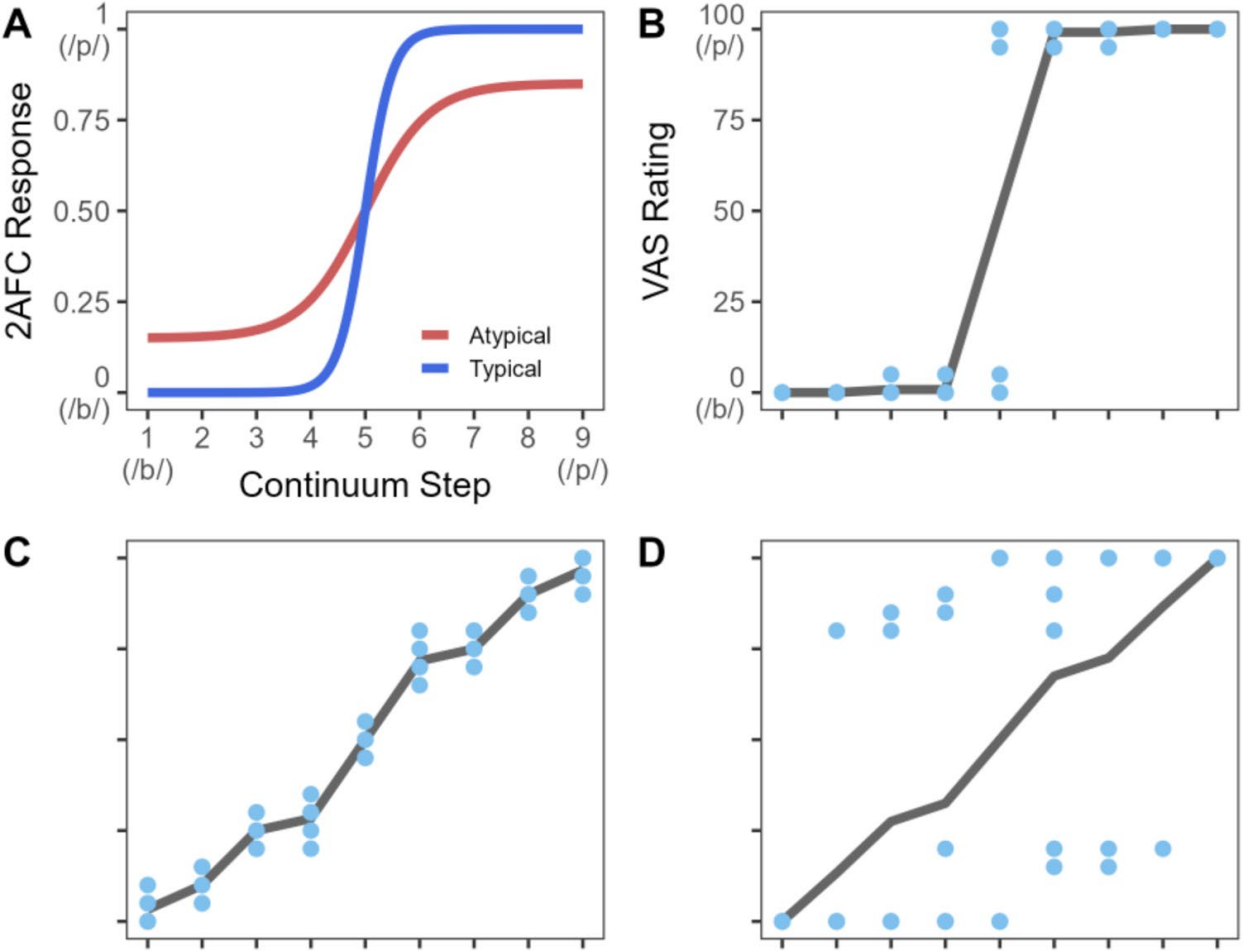
**A** A theoretical plot showing typical responses in forced choice + continuum (2 AFC) task. Continuum Step 1 might represent a /b/ and 9 a /p/. A 0 on the *y*-axis indicates 100% /b/ responding, whereas 1 indicates 100% /p/ responding. Typical listeners (in blue) show a steep, step-like function. Atypical listeners (e.g., with DLD or dyslexia) (in red) show a shallower transition between categories (slope). **B** A hypothetical response from the VAS task. This subject shows a steep mean function. **C** This subject shows a shallow mean function but derives from greater trial-to-trial response consistency (blue dots). **D** This subject shows a similar slope, but one that derives from averaging across low categorization consistency. (Color figure online)

A longstanding method for examining listeners’ mapping between acoustic input and speech categories uses paradigms derived from the early framework of *categorical perception* (Liberman et al., 1957). In these methods, listeners hear tokens from a speech continuum (ranging in small steps between two sounds like /d/ and /t/) and make forced-choice labeling judgments (what we refer to as a forced-choice + continuum task; FC+C). Typically, this is interpreted through a lens in which sharp categorization is “ideal” (a blue line in Fig. 1A), and a psychometric function with a shallower slope indicates noise in the system (a red line in Fig. 1A) (Casillas, 2020; Ziegler et al., 2005). However, two critical problems limit our understanding of the nature of these differences.

First, the psychometric properties of this task are unclear. Very few studies use more than 1–2 speech continua. As a result, from the perspective of individual differences, it is unclear if the shallower slope represents a general disruption of speech categorization or if it is specific to particular types of phonemes or auditory cues (and this could differ in different populations depending on the locus of their differences). Moreover, we have not identified any studies examining any form of reliability in these metrics, and few studies assess the degree to which differences in speech categorization derive from broader factors like cognitive style, rather than auditory processing (though see Yu, 2010; Yu et al., 2013). Thus, while speech categorization is clearly relevant to a range of individual differences, its nature as a trait remains unclear.

Second, FC+C tasks come with significant interpretative limitations—the slope of the categorization function can be ambiguous. Most recent theoretical accounts of speech suggest that gradient categories may be more typical (and could offer benefits to listeners; Kapnoula et al., 2021; Kapnoula & McMurray, 2021; McMurray, 2022; Myers et al., 2024; Toscano et al., 2010). This makes it unclear how to interpret a shallower slope. However, there are also significant psychometric limits.

Consider a listener with an inherently gradient representation—for example, they perceive a sound that is 30% along a continuum as 30% /p/-like. This listener might adopt a decision strategy to always select the most likely response. As a result, even if the sound is only 30% /p/, it could consistently be identified as /b/, producing a steep slope in their categorization function despite the gradient nature of their representation.

Moreover, in the FC+C paradigm, a shallow slope could arise from multiple underlying processes. For example, another listener with the same gradient representation might employ a probability-matching strategy, choosing /p/ 30% of the time and /b/ 70% of the time. This strategy would result in a shallow slope. Conversely, a listener with a discrete underlying representation might display a gradient categorization function if the noise in the system caused the cue value to be miscoded. Consequently, in the FC+C tasks, it is not clear how to disentangle a slope that is shallower due to noise from one that is more gradient. The ability to do so may help distinguish group differences that derive from very different mechanisms and developmental pathways.

### An updated approach to measuring speech categorization

The VAS task (based on Massaro & Cohen, 1983) may provide a richer psychometric approach to speech categorization (for a review, see Apfelbaum et al., 2022). In this task, listeners hear tokens from a speech continuum but select any point on a *continuous* rating scale to indicate the degree of correspondence between the stimulus and either of the two endpoints.

This continuous rating scale allows for a more direct mapping between continuous levels of category activation and the response space which helps to overcome the limits of FC+C tasks. For example, a listener with a steep slope (Fig. 1B) can be easily differentiated from others with a shallower slope. However, two listeners with the same slope may have completely different profiles. If listeners are more gradient, their ratings ought to be linearly related to the continuum step, with any trial-by-trial variance clustered around the mean at that step (Fig. 1C). In contrast, if that shallower mean slope represents noise imposed on a categorical system, we might expect that listeners mostly use the endpoints of the continuum with enhanced variability from trial to trial at the intermediate steps (Fig. 1D). What is critical is the ability to delineate trial-by-trial variability (*categorization consistency*). This cannot be done with forced choice, as the variance is directly proportional to the mean.

Thus, the VAS task offers a more precise delineation of individual differences in speech categorization. Supporting this, a number of studies have linked differences in VAS indices of both slope and consistency to differences in downstream language processing. For instance, individuals with a shallower slope are more sensitive to secondary cues to that phonetic contrast (Kapnoula et al., 2017), and they exhibit a better ability to recover from temporarily misleading information in lexical access (Kapnoula et al., 2021). Recent work has extended this logic to real-world outcomes and predictors like language/reading abilities, perception of second/foreign languages (Fuhrmeister et al., 2023; Honda et al., 2024; Lee & Park, 2024), and the phonetic diversity of talkers in a child’s social network (Kutlu et al., 2024).

As such, the value of VAS as a metric for individual differences is evident. However, it is not yet clear whether it indexes some kind of general trait of overall speech perception ability, and if so, which specific aspects of speech perception might comprise that trait (e.g., slope, consistency). Of course, it is also possible that there is no such trait, that differences in speech perception derive from more stable differences in auditory coding (e.g., temporal vs. spectral cues). Given the common use of speech categorization as a general difference across populations, we started at the more general level, addressing two critical questions. First, what is the nature of this trait? Here we sought any aspect of speech perception that was consistent within a listener across continua. Second, are these differences in VAS responding truly specific to speech, or are they related to broader factors like personality differences or cognitive processing style?

Most prior studies have emphasized the slope of the mean function as indicative of a listener’s long-term category structure. However, it is unclear whether slope is a generally reliable marker of differences. Studies have often found that slope is not consistent across speech continua (e.g., Honda et al., 2024; Kapnoula et al., 2021). For instance, individual slopes in the stop voicing contrast (b/p) were not correlated with those in the fricative place contrast (s/ʃ*)*, suggesting that individual differences in slope may be contrast-specific, rather than a trait (Kapnoula et al., 2021). Critically, most studies have tested a limited range of speech continua, predominantly voicing contrasts in stops, making it difficult to draw firm conclusions about the reliability of slope as an individual trait.

More importantly, recent studies suggest that *categorization consistency* may be a more robust marker of individual speech categorization ability. This index has shown a strong association with success in L2 learners (Fuhrmeister et al., 2023; Honda et al., 2024), and predicted language and reading abilities in children and language abilities in adults, whereas slope was not a critical predictor. However, it is unclear whether it is also consistent across multiple speech continua, or whether consistency derives from broader cognitive factors.

### Speech perception and cognitive processing style

We also ask whether these indices exclusively reflect factors that are specific to speech perception or whether differences may derive from broader cognitive or personality factors. Prior studies have not found a significant relationship between the VAS responses (slope) and domain-general cognitive control, such as working memory or inhibitory control (Kapnoula et al., 2017, 2021), or to parent-report measures of real-world self-regulation. This suggests that domain-general cognitive control may not be a strong predictor of individual differences in speech categorization profiles. The current study extends these prior efforts by testing whether *cognitive processing styles* (e.g., personality traits) can be related to individual differences in speech categorization.

Here, cognitive processing style refers to psychological dimensions that reflect individuals’ preferences or consistencies in how they process information (Ausburn & Ausburn, 1978). Among other factors, autistic traits, or the broader autism phenotypes associated with autism spectrum disorder (ASD), have emerged as predictors of individual variability in speech perception. For instance, the perceptual shift toward existing words (Ganong, 1980) is attenuated in neurotypical individuals with higher levels of autistic traits (Stewart & Ota, 2008) and individuals with higher autistic traits exhibit stronger perceptual normalization for phonetic coarticulation (Yu, 2010). It is not yet clear whether this dimension is related to VAS responding.

Other than autistic traits, there has been limited research on the association between cognitive processing style and speech categorization. Speech categorization involves precise decision-making, and the VAS task in particular may require listeners to tolerate and characterize ambiguity. Thus, it may relate to broader factors such as anxiety or impulsivity. Individuals with anxiety disorders may struggle with decision-making, indecisiveness, and intolerance of uncertainty due to difficulty coping with too many options (e.g., Rassin et al., 2007). In the domain of language processing, speakers with higher anxiety traits show impairments in simple word-production tasks (Snyder et al., 2010, 2014).

Impulsivity, characterized by actions taken without foresight, is also associated with a broader decision-making process (e.g., Zermatten et al., 2005) and is a key component of various psychiatric conditions, including attention-deficit/hyperactivity disorder (ADHD; Winstanley et al., 2006). Individuals with ADHD experience discomfort in perceiving sensory stimuli at lower levels than neurotypical individuals (Fuermaier et al., 2018; Taitelbaum-Swead et al., 2019), and they exhibit impaired speech perception compared to neurotypical listeners (e.g., Blomberg et al., 2019; Derawi et al., 2023; Norrelgen et al., 2007). However, these impairments are typically tied to deficits in attention and working memory capacity rather than the degree of impulsivity. To our knowledge, no research has directly tested the relationship between these general personality factors (anxiety or impulsivity) and low-level speech categorization within a typical range of individuals.

### The present study

The first goal of this study is to ask if each of the two VAS indices—slope or response variability—are consistent properties of individuals. While this can be done in a test/retest reliability framework using the same materials across two test sessions, this cannot address the broad generality of an index—is slope or consistency a marker of a specific difference in specific phonemic contrasts (or auditory dimensions) or of a broader difference in speech categorization?

If a VAS index is indeed a trait-like property of speech categorization, then it should correlate across different phonemic contrasts. Here, by "trait-like," we mean that the VAS index reflects a stable, individual-specific characteristic of speech categorization, which remains consistent across different types of phonemic contrasts. This conceptualization implies that individual differences in categorization (e.g., gradient vs. categorical) may not be confined to a single phonemic contrast but generalize across contrasts, such as stop voicing, vowel, or fricative place distinctions. Specifically, a listener who exhibits gradient categorization in the stop voicing contrast should also show gradient categorization for vowel or fricative place contrasts. This is not to say that listeners may not differ systematically in things like spectral sensitivity that affect only some phonetic contrasts (and these may indeed comprise traits). Rather, our study was not designed to detect such traits.

The second goal of this study is to ask whether individual differences in speech categorization are associated with broader cognitive processing styles: autistic traits, anxiety, and impulsivity as measured by relevant self-reported questionnaires.

## Methods

### Participants

Participants were recruited from an online research platform, Prolific (www.prolific.com). All participants gave informed consent according to the University of Iowa Institutional Review Board requirements and received monetary compensation for their participation ($12 per hour).

A total of 78 participants initially completed all tasks. To ensure the validity of our dataset, we conducted a preliminary examination of raw VAS ratings at the individual level. Participants demonstrating near-flat slopes, which are indicative of random or unreliable responses, were excluded from further analysis. Specifically, the exclusion criterion was applied to individuals whose VAS ratings included greater than 25% of ratings at Step 1 (the extreme lower end) and less than 75% at Step 9 (the extreme upper end), ensuring the exclusion of individuals showing random responses or individuals who were unable to discriminate even the unambiguous words. This data quality check resulted in the exclusion of 10 participants. Consequently, the final sample size included 68 native speakers of American English (33 women) with no history of speech, hearing, or neurological disorders (age mean: 32.2 years, *SD*: 4.9).

### Overall design

The task and questionnaires were built and delivered using Gorilla Experiment Builder (Anwyl-Irvine et al., 2020), and administered using participants’ computers (mobile phones or tablets were not allowed). Before the experiment, participants completed a prescreening block to ensure they wore headphones or earphones; those who failed the prescreening were excluded from further participation. The order and duration of the tasks are summarized in Table 1. Participants completed a VAS task, followed by three questionnaires assessing their anxiety, autistic traits, and impulsivity. Each participant took approximately 45 min to complete the four tasks.

**Table 1:** Order and duration of tasks

Order	Task	Measure	Duration (in min)
1	Visual Analog Scaling (VAS)	Speech categorization	20
2	Penn State Worry Questionnaire (PSWQ)	Anxiety	10
3	Autism-spectrum Quotient (AQ)	Autistic traits	5
4	Urgency-Premeditation-Perseverance-Sensation Seeking-Positive Urgency (UPPS-P)	Impulsivity	10

### Visual analog scaling task

#### Stimuli

Listeners were tested on eight speech continua varying between monosyllabic minimal pairs. Continua spanned the phonetic space, including five vowel pairs (*beet–boot*; *bet–bat*; *hat–hot*; *net–nut*; *pen–pan*), two stop voicing pairs (*beach–peach; dime–time*), and one fricative place pair (*sip–ship*). Some of these were selected to be phonetically close (e.g., there were two ɛ/æ continua and two others that contained one of these phonemes), while others were distal (e.g., s/ʃ vs. b/p), to enable exploratory analyses examining the role of auditory/phonetic proximity in the stability of any underlying traits.

Pairs were recorded by a native male speaker of American English within a carrier sentence, *he said* ____. Target words were excised from the carrier sentence and manipulated into nine steps. We used distinct techniques for each type of continuum that were intended to create the most phonetically valid continua (see Supplement S1 for a discussion). This included using cross-splicing stop voicing continua (McMurray et al., 2008), TANDEM-STRAIGHT for the vowels (Kawahara et al., 1999), and a novel spectral averaging approach developed by Galle et al. (2019) for place of articulation in fricatives (MATLAB code is available at https://osf.io/ut9wz/). Stimuli are available at https://osf.io/36qky/. Detailed descriptions of the stimuli development procedures are available in Supplemental S1.

As all of the continua were derived from natural recordings, we conducted a phonetic analysis to identify the resulting phonetic changes. Table 2 summarizes the acoustic characteristics of the two extreme steps for each contrast type. Each vowel contrast showed considerable differences in terms of either first (F1), second formant frequency (F2), or duration. For the stop voicing contrasts, the *beach–peach* pair showed 41 ms of voice onset time (VOT) differences (the primary acoustic dimension that distinguishes voicing in stops in English; e.g., Francis et al., 2008), while the *dime–time* pair showed even bigger differences (64 ms). Both pairs differed in the duration of the following vowel, a secondary perceptual cue to stop voicing (e.g., Summerfield, 1981). The fricative place contrast (*sip–ship*) showed considerable differences in the spectrum of the frication noise, which is the most critical dimension to distinguish English place contrasts in sibilants (e.g., Jongman et al., 2000). Both the first spectral moment (center of gravity) and the second spectral moment (dispersion or the variance or spread of energy around the mean), showed considerable differences.

**Table 2:** Acoustic characteristics of the baseline tokens

Contrast	Item	F1 (Hz)	F2 (Hz)	F3 (Hz)	Duration (ms)
*A. Vowel contrasts*					
ɛ/æ	pen	648	2011	2750	80
	pan	587	2252	2738	131
ɛ/æ	bet	624	1812	2410	121
	bat	752	1814	2426	193
i/u	beet	274	2544	3197	140
	boot	383	1548	2463	141
æ/ɑ	hat	770	1777	2396	151
	hot	769	1237	2519	143
ɛ/ʌ	net	692	1875	2701	144
	nut	729	1510	2397	127
*B. Stop voicing contrasts*					
		VOT (ms)		Vowel duration (ms)	
b/p	beach	4		146	
	peach	45		107	
d/t	dime	4		295	
	time	68		231	
*C. Fricative place contrasts*					
		Center of gravity (Hz)		Dispersion (Hz)	
s/ʃ	ship	3527		1553	
	sip	8005		1933	

#### Procedure

In each trial of the VAS task, listeners saw a display with a horizontal line along the middle of the screen, with the clipart images of the two endpoint words for one of the continua at either extremity. They then heard an auditory stimulus over a headphone/earphone binaurally and chose a point on the line, signifying how closely the stimulus matched either endpoint. A vertical tick then appeared at their selection point. Listeners could revise their responses before proceeding to the next trial with no time limit. The next trial automatically began at 300 ms after the final response on the previous trial.

Trials were blocked by continua so that listeners would not have to continually remap words to the VAS on each trial. The assignment of phonemes to the side of the continua (e.g., was *peach* on the left or right) was reversed between the two sessions. Each participant completed a total of 432 experimental trials, consisting of nine experimental trials for each continuum with six repetitions (9 steps × 8 continua × 3 reps. × 2 sessions). The presentation order of the continua was randomized within each session. Before the main session, there were three practice trials, consisting of endpoint stimuli for three random pairs. The task took approximately 20 min.

#### Quantifying VAS indices

VAS data is treated as a function relating step number (along the continuum) to VAS response. Data are scaled such that the psychometric function always rises by reversing the VAS scale (e.g., a rating of 100 becomes 0), depending on which word is the endpoint.

**Fig. 2:**
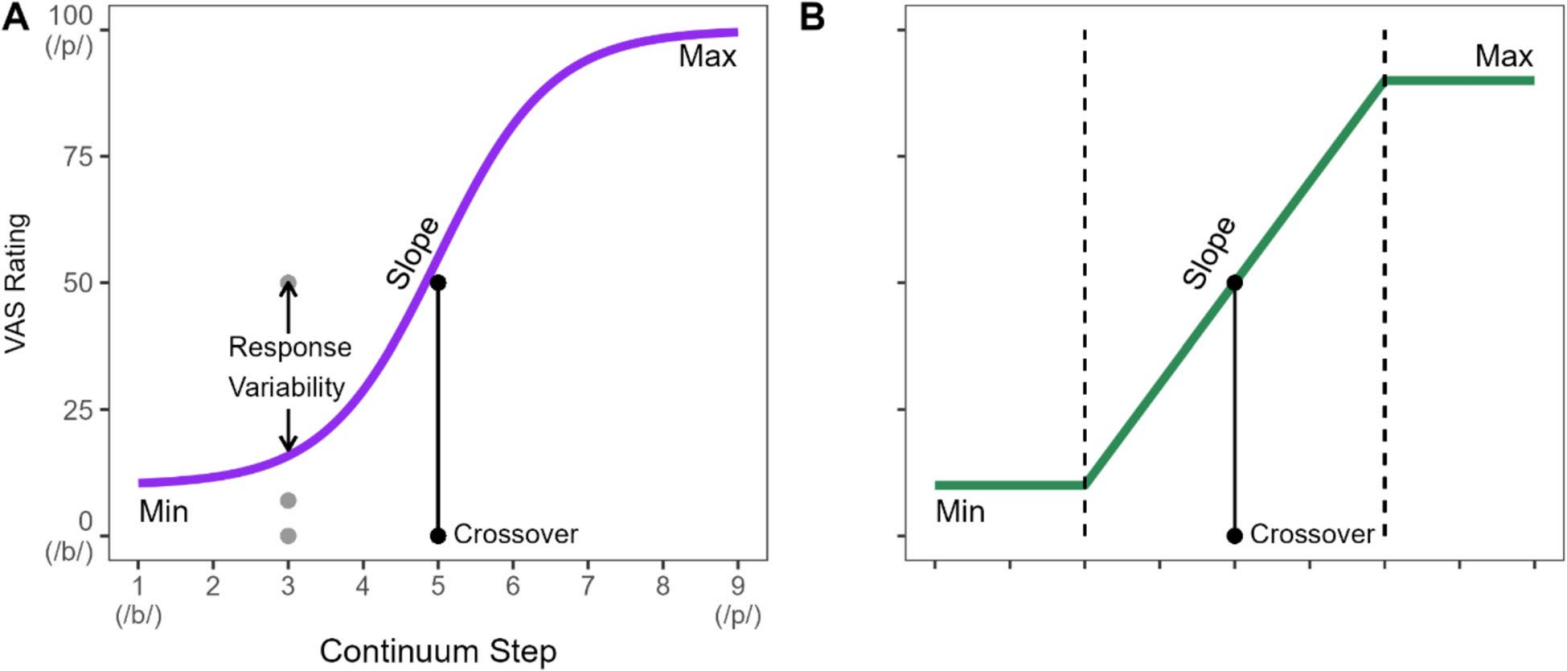
**A** Four-parameters logistic model with response variability. Typical responses in a VAS task of a continuum spanning /b/ (Step 1, 0 on the *y*-axis) to /p/ (Step 9, 100 on the *y*-axis). Dots represent individual trials, and the line is the subjects’ mean. **B** Illustration of the piecewise linear function. Dashed lines are the locations where the growth period of the curve function is located. (Color figure online)

VAS responses are typically modeled with a four-parameter logistic (Fig. 2A). Parameters included the minimum and maximum asymptotes, crossover, and the slope at the crossover point. Sorensen et al. (2024) recently developed such an approach using a non-linear Bayesian mixed model, which could estimate both the overall shape of the VAS responses and individual subjects or individual continuum’s deviations from that pattern (as random effects). However, this model was originally developed for much larger datasets and proved to be difficult to fit into the moderate-sized sample here.

Thus, we developed a variant of that model, which approximated the logistic function with a piecewise linear function. This approximates the logistic function with three lines: two flat lines for the asymptotes (capturing the min and max parameters) and a diagonal between them. The junctures of the flat lines and the diagonals (and the distance between them) capture analogous parameters to the slope and crossover of the logistic. This then captures the same four parameters (see Fig. 2B) but in a less non-linear approach that proved easier to fit.

This function was embedded in a mixed model framework (building on the same Bayesian mixed model framework as Sorensen et al., 2024; Supplement S2 for details), and was implemented in R (R Development Core Team, 2024). The four parameters of the piecewise linear function were implemented as both fixed effects (capturing the overall mean crossover, for example) and with random intercepts for subjects and items. There was a parameter (which appeared as both a fixed intercept and a random intercept for subject and item) that captured the trial-by-trial variance around that function for that subject (response variability).

The Bayesian mixed model approach allows the model to be fit to all the subjects’ data simultaneously, leveraging the full dataset to estimate individual subject/item effects and allowing for differences in the error for each subject to influence the estimated parameters. Moreover, unlike approaches that fit each subject separately, it requires less data/subject. After fitting the full model, we then (post hoc) estimate random intercepts for each subject as each subject’s index of slope, consistency (etc.) for later analysis.^1^ We focused on two key indices.

*Slope* describes how much listeners vary in their perception of tokens within a category and indicates a listener’s longer-term category structure. This parameter is typically thought to reflect the degree to which speech categorization is categorical or gradient: More categorical listeners exhibit steeper slopes, whereas gradient listeners show shallower slopes.

*Response variability* delineates how consistently individual responses map onto categories in the moment of processing. A lower response variability indicates a greater categorization consistency across trials, whereas a higher response variability is indicative of poorer categorization consistency.

### Cognitive processing style measurements

The cognitive processing style and personality measurements consisted of four self-reported questionnaires measuring listeners’ autistic traits, anxiety, impulsivity, and depression.

*Autistic traits* were measured by the Autism-Spectrum Quotient (AQ; Baron-Cohen et al., 2001). This 50-item scale (1 to 4) consists of five components of autistic traits: social skills, attention switching, attention to detail, communication, and imagination. The total AQ and individual component scores were used as predictors, with higher AQ scores indexing a greater presence of autistic traits in individuals.

*Anxiety* was measured by the Penn State Worry Questionnaire (PSWQ; Meyer et al., 1990). It consists of a 16-item scale (1 to 5) evaluating symptoms of anxious apprehension. It is often used for diagnostic purposes, but scores also show good distributions among nonclinical populations. A higher PSWQ score means greater anxiety.

To quantify *impulsivity*, we used Urgency-Premeditation-Perseverance-Sensation Seeking-Positive Urgency (UPPS-P; Lynam et al., 2006). The UPPS-P consists of a 59-item scale (1 to 4) evaluating factors that could lead to individuals’ impulsive behaviors. There are five subcomponents, assessing participants’ tendency to experience strong impulses under negative conditions (negative urgency), tendency toward rash action under positive mood (positive urgency), tendency to fail to think before engaging in an act (premeditation), difficulties focusing on a task (perseverance), and tendency to pursue exciting activities (sensation seeking). A higher UPPS-P score indicates greater impulsivity.

Participants completed these questionnaires after the VAS task. Each questionnaire took less than 10 min to complete.

## Results

**Fig. 3:**
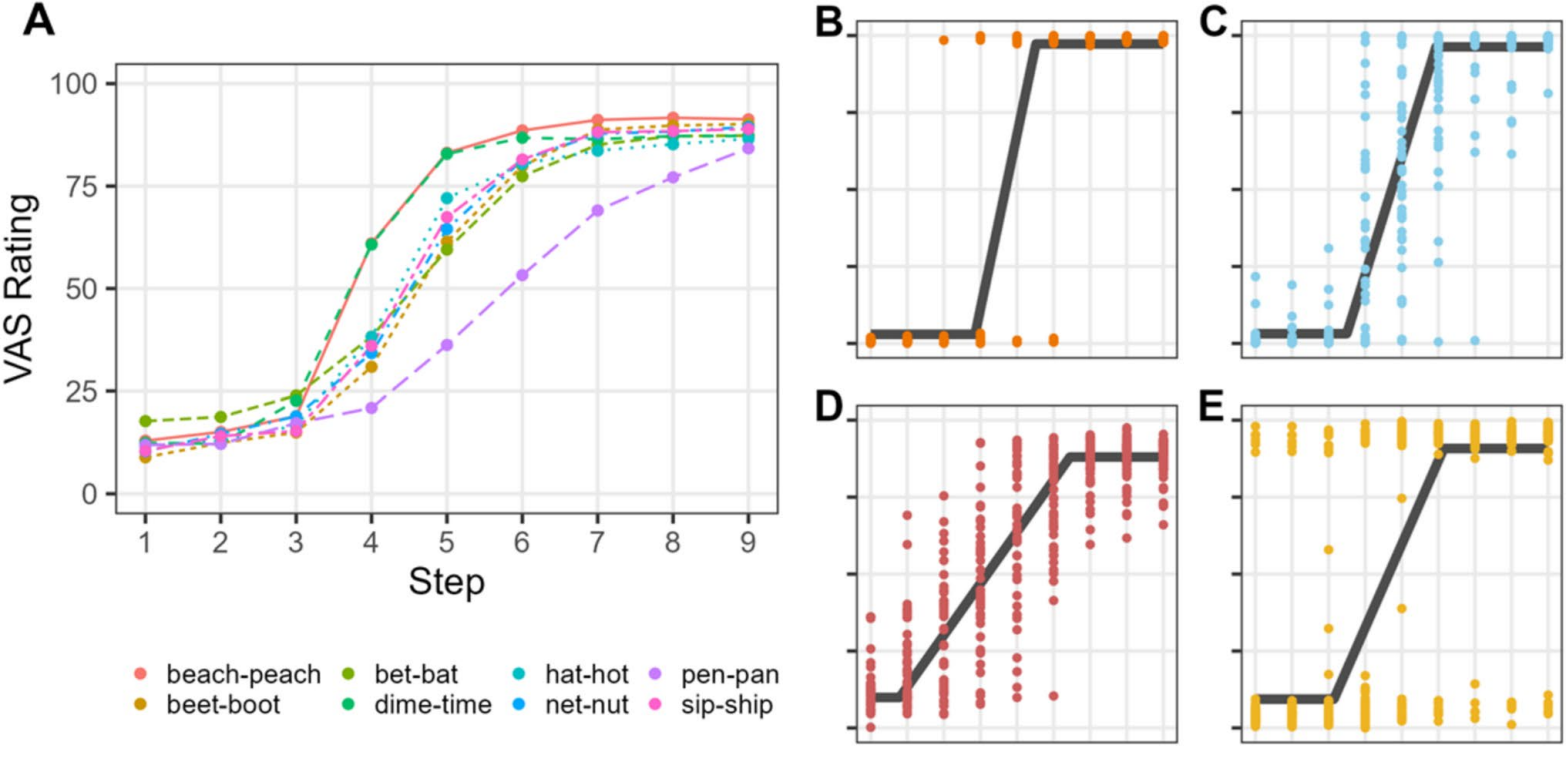
**A** VAS ratings as a function of continuum step and phonological contrast. For individual plots shown in B–E, each dot is a subject’s response on a given trial, and lines are logistic curves fit to responses. **B** VAS ratings by a listener who mostly uses the endpoints, showing a steep slope. **C** Responses by a subject who exhibits a similar slope to B, but this slope is derived from more flexible use of the VAS scale. **D** A listener with a shallow slope with relatively high categorization consistency. **E** A subject with a similar slope to D, but with lower consistency due to more use of the endpoints. (Color figure online)

Figure 3A illustrates the overall VAS ratings as a function of the continuum step and continuum type. At the group level, stop voicing contrasts (*beach–peach* and *dime–time*) exhibited visually steeper slopes than vowel and fricative place contrasts. Among the vowel contrasts, the *pen–pan* contrast showed the shallowest slope.

As shown in Fig. 3B–E, individual listeners varied markedly in their speech perception gradiency. Even when two listeners presented similar mean slope functions, their trial-by-trial response consistency could differ substantially. For instance, the listener shown in Fig. 3B predominantly used the extreme endpoints, whereas the listener depicted in Fig. 3C employed the entire VAS scale. Despite both listeners showing similarly steep slopes, these slopes derived from distinctly different trial-by-trial responses. Likewise, the listeners shown in Fig. 3D and E show similarly shallower slopes (relative to Fig. 3B and C). However, the listener in Fig. 3D displayed more consistent trial-by-trial responses, closely clustering around the mean function, whereas the listener in Fig. 3E showed noisier trial-to-trial responses, mostly using the extreme points of the scale. These differences in response variability underscore the necessity of examining response consistency in accounting for individual differences in speech categorization.

We thus extracted slope and response variability for each listener and each continuum from the Bayesian model. We then examined the correlation (across subjects) for each pair of continua using R (Version 4.4.2; R Development Core Team, 2024). We did not assess the statistical significance of individual correlations as our study did not aim to draw strong conclusions from individual correlations but rather to interpret trends across multiple correlations, emphasizing effect sizes over isolated statistical significance. However, we report the full matrix in Supplement S3 (at *N* = 68, the critical value is *r* >.239).

**Fig. 4:**
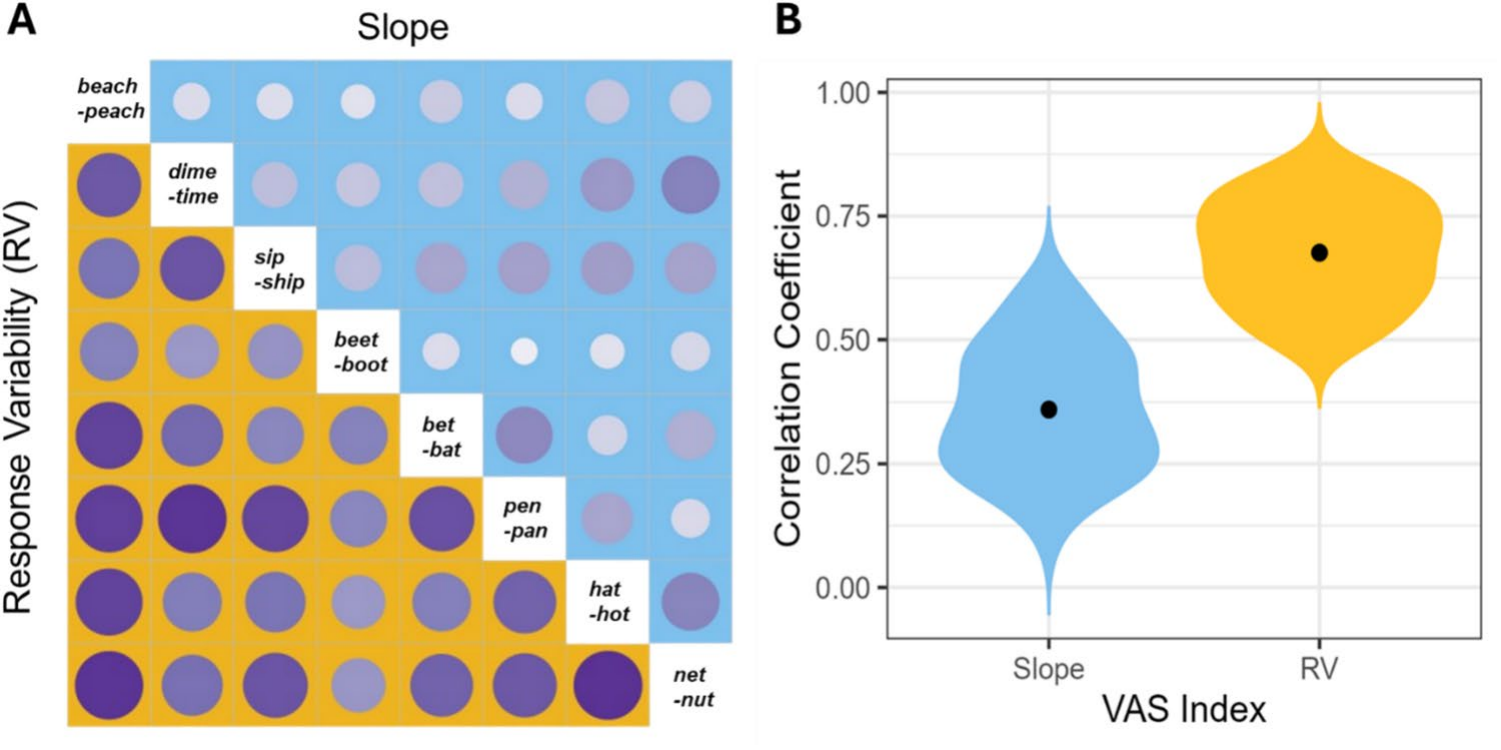
**A** Pair-wise correlations between continua. The cells above the diagonal (in blue) reflect slope, and those below the diagonal (in yellow) represent response variability. The bigger and darker the circle in each cell, the higher the correlation coefficients. **B** Summary of correlations between continua as a function of VAS indices. The black dots indicate the mean coefficient of each index. The length of the violins indicates the range of coefficients, and the width at a given *y* value represents the point density at that value. (Color figure online)

The correlation matrix is illustrated in Fig. 4A. Across all continua, response variability (lower yellow panels) showed strong positive correlation coefficients: a participant with low variability on the *beach–peach* continuum was also likely to have low variability on other continua (etc.). In contrast, slope (upper blue panels) exhibited relatively weaker correlations. Consistency was more “consistent” across continua than slope, with average correlations for consistency (*r* =.67) nearly twice those of slope (*r* =.36; Fig. 4B).

To examine the relationships between VAS indices and other cognitive factors, we next conducted multiple regressions predicting average slope and response variability from the cognitive processing factors. Given the significant conceptual overlap among the predictors (e.g., correlations between AQ and PSWQ), a multiple regression approach was employed to assess the unique contributions of each trait to the dependent variables after accounting for the effects of the others. This approach allows for a more nuanced understanding of the predictors’ individual and joint effects on categorization performance.

Prior to the regression, we examined correlations among the variables to guard against collinearity (see Supplement S4 for a full correlation matrix). Among personality traits, AQ scores were positively correlated with PSWQ scores, *r* = 0.55, *p* <.001, suggesting that individuals with higher autistic traits tend to have a greater degree of anxiety. The UPPS scores did not show significant correlations with other measures, indicating that impulsivity traits are not associated with autistic traits or anxiety.

The first regression model assessed the relationship between the categorization slope and three independent variables: PSWQ, UPPS, and AQ. The model (Table 3A) was not statistically significant, *F*(3, 64) = 1.02, *p* =.39, with an adjusted *R*^2^ = 0.001, indicating that the predictors collectively explained less than 1% of the variance in slope. None of the predictors was individually significant.

**Table 3:** Summary of coefficients in the regression models on VAS indices, with *z*-scored PSWQ, UPPS, and AQ scores as fixed effects

Fixed effects	Estimate	*SE*	*t*	*Pr*(>|t|)
**A. Slope**				
(Intercept)	− 0.038	0.123	− 0.308	.759
PSWQ	0.173	0.153	1.132	.262
UPPS	− 0.178	0.122	− 1.459	.150
AQ	− 0.053	0.151	− 0.350	.728
**B. Response variability**				
(Intercept)	0.003	0.126	0.023	.982
PSWQ	− 0.031	0.157	− 0.199	.843
UPPS	0.004	0.124	0.030	.976
AQ	0.056	0.154	0.361	.719

*Note.* R syntax: lm(VAS.Index.Z ~ PSWQ.Z + UPPS.Z + AQ.Z)

The second regression model asked whether the same independent variables predicted response variability. This model (Table 3B) was also not statistically significant, *F*(3, 64) = 0.04, *p* =.99, with an adjusted *R*^2^ = − 0.045, suggesting the predictors explained negligible variance in response variability. As with the first model, none of the predictors were statistically significant. These results do not provide evidence for a significant predictive relationship between anxiety, impulsivity, autistic traits, and VAS indices.

One concern with this is that if the average slope (across continua) for a subject is not reliable, it would not be expected to correlate with subject-level descriptors. Thus, we conducted a simple correlation analysis that asked whether autistic traits or personality measures relate to the categorization slope and consistency for individual phonological contrasts (rather than the average). However, there were no significant relationships between these variables (see Supplement S4 for the complete results). Taken together, it is less likely that there is an association between higher cognitive processing style and low-level speech categorization at an individual level, at least for the general cognitive factors that we measured for this study.

## Discussion

This study sought to identify the dimension(s) of speech categorization that most effectively capture individual differences in speech categorization. We addressed this question using the VAS task, which offers a unique dimensionalization of the problem by separately characterizing the slope and the trial-by-trial variability. It was used with a large set of speech continua, including stop voicing, vowel, and fricative place contrasts. We found that speech categorization consistency—reflecting trial-by-trial response variability—is a highly stable, trait-like property of individuals across all continua. Importantly, categorization consistency was correlated across continua at roughly twice the rate of slope. Thus, categorization consistency was a more robust and stable index than slope, which has typically been regarded as the primary index for assessing individual differences in speech perception.

It is unclear what developmental factors lead to the stability of categorization consistency. Work in progress suggests that stability is a clear locus of development up through at least 6 th grade (Kutlu et al., 2025), with children becoming increasingly more consistent with age. One possibility is that categorization consistency derives from neural differences in the encoding of sounds in general (e.g., in the auditory nerve and subcortical tracts; Hornickel & Kraus, 2013; Skoe et al., 2015). However, developmental work suggests encoding variability stabilizes well before this period. There may also be higher-level clean-up mechanisms that stabilize perception, but that are based on more general properties (e.g., phonological regularities, lexical structure) that are more sensitive to things like overall language ability or the quality of the language learning environment and are thus less specific to each cue.

While categorization consistency proved to be a more stable metric than slope, this does not necessarily mean that slope is not useful in characterizing individual speech categorization profiles. However, it may be more specific to how particular categories, structures, or acoustic cues are encoded (e.g., Kapnoula & McMurray, 2021). Prior work clearly shows, for example, that individuals who are more gradient in a stop voicing contrast exhibit differences in neural encoding of voicing cues, as well as in cue integration and downstream lexical processing. However, this difference may not be relevant for fricatives, which rely on different auditory or phonetic cues. Thus, the slope of a given contrast matters for how that contrast is used.

This raises two possibilities. First, slope may be more associated with more auditory level individual differences like spectral or temporal fidelity that are highly relevant for some issues (e.g., hearing impairment). Indeed, this may be useful in hearing loss, where interventions like cochlear implants may drive reliable differences in spectral sensitivity. Second, it is now well known that the “structure” of the category (reflected in the slope) may be tuned to the distribution of phonetic cues in the language learning environment (Kutlu et al., 2024; Maye et al., 2002; Theodore & Monto, 2019; Theodore et al., 2015). Such distributions may be specific to the talkers of the environment and less stable across contrasts (e.g., a talker with more discrete voicing categories may not have more discrete vowel categories). However, either way, our work suggests slope may not be a robust marker of speech perception in general (as it is often used in work on clinical populations).

While we have focused on overall trait-like qualities that extend across continua, some aspects of speech perception may reflect person-specific auditory differences that are specific to a single or a set of related contrasts. This may be important for some real-world differences—for example, cochlear implant users, who typically struggle more with transmitting spectral details than temporal ones (Nie et al., 2006; Wilson & Dorman, 2008), may exhibit important trait-like differences that are specific to certain cues (e.g., VOT, manner of articulation) but not to others (e.g., vowel formants). We did not observe that here—our two VOT continua, for example, were not better correlated in consistency (*r* =.71) than they were with other contrasts (*r* =.72), and a similar effect was observed for slope (*r* =.24 vs. *r* =.35). In fact, such differences may be better captured by slope than consistency.

Nonetheless, our design did not attempt to systematically examine this, and it seems likely that categorization indices (either slope or consistency) might capture finer-grained aspects of speech perception in addition to the general traits we see in consistency. This limitation is acknowledged as a potential avenue for future research. Incorporating a broader range of contrasts sensitive to different acoustic dimensions, such as temporal, spectral, and amplitude information, could further clarify the interaction between general traits and contrast-specific processing.

Equally important to our findings on categorization consistency, we did not observe a significant association between individuals’ categorization profiles and their cognitive processing styles. When coupled with prior findings showing similar null results for cognitive control (Kapnoula et al., 2017, 2021) and self-regulation, this finding suggests that VAS responses are less likely to be affected by higher cognitive demands and decision-making processes and may reflect processes more specific to speech and language. This is underscored by a recent study showing that only the categorization consistency in speech robustly accounts for the variance in overall language ability, even after controlling for categorization consistency in a closely matched visual task. Together, this evidence suggests that categorization consistency is more likely to be specific to speech perception rather than task-related or other cognitive processes.

Despite evidence suggesting that VAS indices are independent of broader cognitive differences, the underlying mechanisms of consistency remain poorly understood. Specifically, while categorization consistency has emerged as a stable, trait-like property of individuals and a robust predictor of downstream language processing and real-world outcomes, its fundamental dimensionality remains unclear. Given that we have only examined a small number of factors involved in general cognitive processes, it would be premature to make a firm conclusion that categorization consistency is unique to speech perception, and in fact, categorization consistency in other domains (e.g., face perception) may also be trait-like and relevant for other outcomes (e.g., autism, social function). Further investigation is needed to determine whether this consistency is speech-specific, auditory, or domain-general.

From a theoretical perspective, our results highlight the need for an updated theoretical model of speech perception that incorporates categorization consistency. Traditional models of speech perception have emphasized category *representations* (Goldinger, 1998; Kronrod et al., 2016; McClelland & Elman, 1986; McMurray & Jongman, 2011; Oden & Massaro, 1978). However, our results, along with recent evidence, suggest that the degree to which the perceptual system consistently arrives at the same interpretation of the input is a critical factor. This aspect is not adequately captured by theoretical models of speech perception.

Empirically, our findings underscore the importance of including diverse speech continua in speech categorization research. Much of the existing work on individual differences in the speech VAS task has relied on a limited range of speech continua, raising questions about the generalizability of these findings (particularly if slope is the primary predictor) across different types of speech continua. Our continuum analysis demonstrated that an individual’s categorization slope may not be consistent across all types of continua, suggesting that prior findings regarding categorization slope may not hold when replicated with different speech continua. In contrast, categorization consistency may serve as a more stable metric for individual differences in speech perception.

## Supplementary Information

Below is the link to the electronic supplementary material.

Supplementary file1 (DOCX 70 KB)

## Data Availability

Data and materials of the present study are available at an Open Science Framework site for this study (https://osf.io/36qky/).

## Footnotes

1 Note that in principle it should have been possible to include individual difference variables (e.g., impulsivity) as between-subject predictors of the parameters of this model. This was not done for two reasons. First, this added a large number of free parameters (4 predictors × 5 parameters) that made the model more difficult to fit. Second, it precluded the ability to use tools like commonality analysis to identify unique and shared variance (which a priori we suspected would be necessary).

## Code availability

Codes of the present study are available at an Open Science Framework site for this study (https://osf.io/36qky/).
