# Supplementary material for: The consistency of categorization-consistency in speech perception
Source: Psychon Bull Rev. 2025 Apr 24;32(5):2246–58. doi: 10.3758/s13423-025-02700-x (PMC12426149; doi:10.3758/s13423-025-02700-x)
Supplement: Supplementary file 1 — Supplementary file1 (DOCX 70 KB) [file 13423_2025_2700_MOESM1_ESM.docx]

**The Consistency of Categorization-Consistency in Speech Perception**

**Supplementary Materials**

**Hyoju Kim ^a^, Bob McMurray ^a, b^, Eldon Sorensen ^c^, Jacob Oleson ^d^**

^a^ Department of Psychological & Brain Sciences, University of Iowa, Iowa City, IA 52242, USA

^b^ Department of Linguistics, University of Iowa, Iowa City, IA 52242, USA

^c^ Sandia National Laboratories, Albuquerque, NM 87123, USA

^d^ Department of Biostatistics, University of Iowa, Iowa City, IA 52242, USA

* Corresponding author: Hyoju Kim, Department of Psychological & Brain Sciences, University of Iowa, PBSB 255E, Iowa City, IA, 52242, USA.

E-mail addresses: hyoju-kim@uiowa.edu

**Contents**

S1. Details of Stimulus Construction

S2. Details of Statistical Modeling

S3. Correlations between Continua

S4. Correlations between VAS Indices and Cognitive Factors

**S1. Details of Stimulus Construction**

This study used eight speech continua: two stop voicing continua (*beach-peach* and *dime-time*), a fricative place continuum (*sip-ship*), and five vowel continua (*beet-book, bet-bat, pen-pan, hat-hot,* and *net-nut*). These were all based on manipulations of natural recordings of a real talker.

The selection of the specific methods used to create each contrast was driven by the specific acoustic properties required to instantiate that phonetic distinction. While in principle, a tool like TANDEM-STRAIGHT (Kawahara et al., 1999) could have been used for all contrasts, the use of specialized techniques for each phonetic dimension allowed us to preserve the ecological validity of the stimuli. This tailored approach minimized the risk of introducing methodological artifacts and ensured that the contrasts reflected the natural acoustic/phonetic dimensions central to perception.

TANDEM-STRAIGHT performs particularly well with *spectral* manipulations in vocalic portions of the stimuli, where it can estimate pitch and formants. However, for other types of contrasts, it can yield perceptually unnatural outputs. As vowel quality is primarily determined by formant structure, TANDEM-STRAIGHT was the most effective tool for manipulating the relevant cues. In contrast, stop voicing relies on temporal cues (VOT), which can be manipulated more naturally by cross-splicing natural productions. While the fricative place contrast is primarily governed by spectral properties, the aperiodic nature of the frication was more difficult for TANDEM-STRAIGHT and could be better manipulated through a spectral averaging we developed in prior work (Galle et al., 2019).

We started by recording each endpoint word (e.g., *beach* and *peach*), spoken by an adult male English speaker with an American Midwestern accent. These words were recorded in a carrier sentence, “*He said ____*” to ensure a consistent prosody and rate across the words. We then selected one exemplar to serve as the endpoint for each continuum, seeking exemplars that were spoken with a uniform falling prosody and were free of any artifacts. Once we had selected the two endpoint tokens for each continuum, we used continuum-specific methods to create a 7-step continuum.

The ***stop voicing*** contrasts (*beach-peach* and *dime-time*) manipulated VOT using a progressive cross-splicing procedure (Andruski et al., 1994; McMurray et al., 2008) using Praat (Boersma & Weenink, 2021). We selected segments of the aspiration from /p/ and /t/ whose duration corresponded to the intended VOT and then replaced the corresponding quantity of the onset of /b/ and /d/ (respectively) with this aspiration, thus approximately maintaining the overall length of the word, but with less vocalic energy and more aspiration at word onset for voiceless sounds.

To create the vowel contrasts, we used TANDEM-STRAIGHT (Kawahara et al., 1999) (a MATLAB tool) which first extracts periodic information for each endpoint. Next, temporal anchors are manually placed at the beginning, middle, and end of the target sounds. Finally, continua were morphed across two endpoints in nine steps. This approach allows for precise control over formant frequencies, enabling us to interpolate between vowel endpoints while maintaining naturalness in pitch and duration. We chose this for vowel continua because it excels in preserving the fine-grained acoustic details necessary for distinguishing vowel quality, which is primarily encoded in formant structure.

For the fricative contrast, we employed a spectral averaging procedure that was developed for prior studies (Colby et al., 2023; Galle et al., 2019; McMurray et al., 2018). First, the frication portions of the endpoint tokens (*sip* and *ship*) were extracted from the selected recordings. Second, the longer frication segment was cut to match the length of the shorter one, ensuring consistency in segment length. Third, the overall spectral mean was calculated from the long-term average spectra of each fricative, and both spectra were aligned to the same average spectral mean. Fourth, a weighted average of the two spectra was constructed to create a series of nine spectra, representing each step along the continuum. Fifth, the spectral means of the spectra were shifted in frequency space to create nine steps. Sixth, the modified spectra were applied as filters to a segment of white noise, which had an envelope that was the average of the /s/ and /ʃ/ endpoints. Lastly, we spliced the vocoid from the original recording of /s/. This procedure was implemented using MATLAB (<https://osf.io/ut9wz/>).

One concern is that differences in stimulus generation methods could affect the stability of VAS indices across continua. For example, categorization of a vowel continuum is more consistent with other vowels (which were also made with TANDEM-STRAIGHT) than with a voicing continuum (made with cross-splicing). However, in our view, the goal of attaining more phonetically realistic dimensions was more appropriate, which led us to use the most established and contrast-appropriate technique for each type of continuum. This rationale ensures that differences in perceptual patterns across continua are more likely attributable to the phonetic contrasts themselves rather than methodological inconsistencies.

Moreover, it did not appear to be the case that stimulus construction drove these results. The five TANDEM-STRAIGHT-generated continua were not more correlated with each other than they were with other continua for either slope (STRAIGHT with STRAIGHT: *r* = .34; STRAIGHT with other: *r* = .39) or consistency (STRAIGHT with STRAIGHT: *r* = .65; STRAIGHT with other; RV: *r* = .69). Additionally, the two VOT continua were not significantly more correlated with each other than they were with other continua for either slope (VOT with VOT: *r* = .24; VOT with other: *r* = .35) or consistency (VOT with VOT: *r* = .72; VOT with other RV: *r* = 0.71).

**S2. Details of Statistical Modeling**

The VAS response was assumed to be normally distributed $Y_{ijk}\sim N\left( \mu_{ij},\sigma_{ij}^{2} \right)$ with $i$ denoting subject, $j$ denoting a VAS step, and $k$ denoting trial. The mean follows a four-parameter piecewise-linear approximation (4-PPLA) which includes four parameters to approximate the four-parameter logistic function. These are the minimum, maximum, and two knots ($k_{1},k_{2}$) that describe the transition from the minimum to the maximum (dotted lines in Figure 2B). The equation is:

$\mu_{ijk}=\beta_{1}+slope*\left( step-k_{1} \right)*I\left( step>k_{1} \right)+\left( -slope \right)*\left( step-k_{2} \right)*I(step>k_{2})$ (1)

Let $\beta_{1}$ and $\beta_{2}$ represent the minimum and maximum, respectively, and define *k_1_* and *k_2_* as

$k_{1}=\beta_{3}-\beta_{4}$ (2)

$k_{2}=\beta_{3}+\beta_{4}$.

The $\beta_{3}$ and $\beta_{4}$ parameters are defined to further allow the 4-PPLA to mirror the 4-parameter logistic function with $\beta_{3}$ being the inflection point midway between the knots $k_{1}$ and $k_{2}$. $\beta_{4}$ is then the distance between the inflection point, and the knot. The slope can then be calculated as a function of these parameters with y change divided by x change such that slope = $\frac{\beta_{2}-\beta_{1}}{2\beta_{4}}$.

The minimum and maximum parameters (*β_1_* and *β_2_*) each had a random intercept for subject. We initially experimented with a random intercept of subject for the knots (*k_1_* and *k_2_*). However, estimating those within the model turned out to be prohibitively slow. Instead, subject-specific knots were estimated prior to fitting the full model and fixed. Each parameter had random intercepts of *continuum*, and *subject × continuum* (deviances that were specific to that subject for that continuum).

The variance, $\sigma_{ij}^{2}$, was estimated using a log-linear model with a linear and quadratic effect of continuum step, an effect of that subject’s slope (estimated from the other parameters), and a random intercept for *subject*, *continuum*, *and subject x continuum* (equation 3). Thus, the variance around the predicted function for each individual depends on their own slope as well as the location along the continuum (since variance is predicted to be higher toward the center of the function). Here, *B_0-3_* _are_ free parameters, slope is estimated from the knot points and the asymptotes.

$\log\left( \sigma^{2} \right)\sim B_{0}+B_{1}\cdot step+B_{2}\cdot{step}^{2}+B_{3}\cdot slope$ (3)

Estimating all of these parameters simultaneously within the full Bayesian model was doable but took three times longer to fit relative to the 4-parameter logistic (145 hours versus 58 hours). In order to make the model more efficient, we estimated the knots using the R package *saemix* (Comets et al., 2017) prior to fitting the Bayesian model that estimated the slope. A comprehensive simulation study verified that this approach yielded reasonable accuracy and coverage of the inflection point and slope.

Vague normal priors were given to the parameters necessary to complete the model.

**S3. Correlations between Continua**

Table S3 presents correlations between continua for each of the VAS indices, with Table S3A displaying the correlations for slope and Table S3B for response variability.

| **Table S3.** Correlations between continua. |
| --- |

**A.** Summary of correlation coefficients for slope

|  | 1 | 2 | 3 | 4 | 5 | 6 | 7 |
| --- | --- | --- | --- | --- | --- | --- | --- |
| 1. beach-peach | 1 |  |  |  |  |  |  |
| 2. dime-time | .24 | 1 |  |  |  |  |  |
| 3. sip-ship | .23 | .36 | 1 |  |  |  |  |
| 4. beet-boot | .20 | .34 | .37 | 1 |  |  |  |
| 5. bet-bat | .32 | .35 | .46 | .23 | 1 |  |  |
| 6. pen-pan | .24 | .42 | .48 | .12 | .57 | 1 |  |
| 7. hat-hot | .33 | .50 | .49 | .21 | .27 | .46 | 1 |
| 8. net-nut | .30 | .59 | .47 | .26 | .43 | .26 | .58 |

**B.** Summary of correlation coefficients for categorization consistency

|  | 1 | 2 | 3 | 4 | 5 | 6 | 7 |
| --- | --- | --- | --- | --- | --- | --- | --- |
| 1. beach-peach | 1 |  |  |  |  |  |  |
| 2. dime-time | .72 | 1 |  |  |  |  |  |
| 3. sip-ship | .65 | .73 | 1 |  |  |  |  |
| 4. beet-boot | .60 | .51 | .53 | 1 |  |  |  |
| 5. bet-bat | .79 | .68 | .57 | .60 | 1 |  |  |
| 6. pen-pan | .80 | .83 | .78 | .57 | .75 | 1 |  |
| 7. hat-hot | .79 | .61 | .64 | .51 | .61 | .70 | 1 |
| 8. net-nut | .82 | .65 | .74 | .51 | .69 | .72 | .83 |

**S4. Correlations between VAS Indices and Cognitive Factors**

Table S4.1 provides a summary of the correlations between VAS indices and cognitive processing factors. We applied the Bonferroni correction specifically to a subset of correlations, which included comparisons between each VAS index and each of the cognitive processing factors. The uncorrected *p*-values showed a significant correlation slope and PSWQ (*p* < .05). However, after applying the correction, the significance threshold was adjusted to a more stringent alpha level (*α* = 0.0083). The corrected *p*-value for the correlation between slope and PSWQ was no longer significant.

| **Table S4.1.** Correlation matrix of the study variables | | | | | |
| --- | --- | --- | --- | --- | --- |
| Variable | 1 | 2 | 3 | 4 | |
| 1. Categorization slope | 1 |  |  |  | |
| 2. Categorization consistency | −.46*** | 1 |  |  | |
| 3. AQ | −.01 | .05 | 1 |  | |
| 4. PSWQ (Anxiety) | .07 | .03 | .55*** | 1 | |
| 5. UPPS (Impulsivity) | −.16 | −.05 | .11 | .15 | |
| *Note*. For the unshaded correlations, *** *p* < .001. For the shaded correlations, *p-*values are Bonferroni corrected to *α* = 0.0083. | | | | |  |

Table S4.2 reports correlations between VAS indices and cognitive processing factors for each continuum. These were not family-wise error-corrected as the goal was exploratory: if any of these had a reasonable effect size, this might warrant a deeper analysis.

| **Table S4.2.** Relationship between VAS indices and cognitive processing style as a function of continuum type. |
| --- |

**A.** Summary of correlation coefficients between slope and cognitive processing style

| **Contrast** | **AQ** | **PSWQ** | **UPPS** |
| --- | --- | --- | --- |
| *beach-peach* | .003 | .09 | .01 |
| *dime-time* | −.08 | .09 | −.23 |
| *sip-ship* | −.05 | .09 | .03 |
| *beet-boot* | .05 | −.01 | −.02 |
| *bet-bat* | .15 | .15 | −.2 |
| *pen-pan* | .18 | .25 | −.1 |
| *hat-hot* | −.07 | .07 | .04 |
| *net-nut* | −.06 | .06 | −.04 |

**B.** Summary of correlation coefficients between categorization consistency and cognitive processing style

| **Contrast** | **AQ** | **PSWQ** | **UPPS** |
| --- | --- | --- | --- |
| *beach-peach* | .02 | .01 | −.04 |
| *dime-time* | .02 | −.07 | .08 |
| *sip-ship* | .05 | .09 | −.01 |
| *beet-boot* | −.09 | .03 | .11 |
| *bet-bat* | −.05 | −.08 | .04 |
| *pen-pan* | −.08 | −.05 | −.04 |
| *hat-hot* | .04 | −.03 | −.09 |
| *net-nut* | .12 | .08 | −.11 |

**References**

Andruski, J. E., Blumstein, S. E., & Burton, M. (1994). The effect of subphonetic differences on lexical access. *Cognition*, *52*(3), 163-187. <https://doi.org/10.1016/0010-0277(94)90042-6>

Boersma, P., & Weenink, D. (2021). *Praat: doing phonetics by computer*. In (Version 6.1.42) <http://www.praat.org/>

Colby, S., Seedorff, M., & McMurray, B. (2023). Audiological and Demographic Factors that Impact the Precision of Speech Categorization in Cochlear Implant Users. *Ear Hear*, *44*(3), 572-587. <https://doi.org/10.1097/AUD.0000000000001307>

Comets, E., Lavenu, A., & Lavielle, M. (2017). Parameter Estimation in Nonlinear Mixed Effect Models Using saemix, an R Implementation of the SAEM Algorithm. *Journal of Statistical Software*, *80*(3). <https://doi.org/10.18637/jss.v080.i03>

Galle, M. E., Klein-Packard, J., Schreiber, K., & McMurray, B. (2019). What Are You Waiting For? Real-Time Integration of Cues for Fricatives Suggests Encapsulated Auditory Memory. *Cogn Sci*, *43*(1), e12700. <https://doi.org/10.1111/cogs.12700>

Kawahara, H., Masuda-Katsuse, I., & de Cheveigné, A. (1999). Restructuring speech representations using a pitch-adaptive time-frequency smoothing and an instantaneous-frequency based F0 extraction: Possible role of a repetitive structure in sounds. *Speech Communication*, *27*(3), 187-207. <https://doi.org/https://doi.org/10.1016/S0167-6393(98)00085-5>

McMurray, B., Aslin, R. N., Tanenhaus, M. K., Spivey, M. J., & Subik, D. (2008). Gradient sensitivity to within-category variation in words and syllables. *J Exp Psychol Hum Percept Perform*, *34*(6), 1609-1631. <https://doi.org/10.1037/a0011747>

McMurray, B., Danelz, A., Rigler, H., & Seedorff, M. (2018). Speech categorization develops slowly through adolescence. *Dev Psychol*, *54*(8), 1472-1491. <https://doi.org/10.1037/dev0000542>
